# Why we must question the militarisation of conservation

**DOI:** 10.1016/j.biocon.2019.01.013

**Published:** 2019-04

**Authors:** Rosaleen Duffy, Francis Massé, Emile Smidt, Esther Marijnen, Bram Büscher, Judith Verweijen, Maano Ramutsindela, Trishant Simlai, Laure Joanny, Elizabeth Lunstrum

**Affiliations:** a University of Sheffield, United Kingdom; b Institute of Social Studies, The Hague, Netherlands; c University of Ghent, Belgium; d University of Cape Town, South Africa; e University of Cambridge, United Kingdom; f York University, Canada

**Keywords:** Anti-poaching, Human-wildlife conflict, Militarisation, Poaching, Political economy, Protected areas, Rangers, Trafficking, Violence, Wildlife trade

## Abstract

Concerns about poaching and trafficking have led conservationists to seek urgent responses to tackle the impact on wildlife. One possible solution is the militarisation of conservation, which holds potentially far-reaching consequences. It is important to engage critically with the militarisation of conservation, including identifying and reflecting on the problems it produces for wildlife, for people living with wildlife and for those tasked with implementing militarised strategies. This Perspectives piece is a first step towards synthesising the main themes in emerging critiques of militarised conservation. We identify five major themes: first, the importance of understanding how poaching is defined; second, understanding the ways that local communities experience militarised conservation; third, the experiences of rangers; fourth, how the militarisation of conservation can contribute to violence where conservation operates in the context of armed conflict; and finally how it fits in with and reflects wider political economic dynamics. Ultimately, we suggest that failure to engage more critically with militarisation risks making things worse for the people involved and lead to poor conservation outcomes in the long run.

## Highlights

• Draws together key themes in the critical literature in militarisation
• Shows why the expansion of militarisation needs to be challenged
• Argues militarisation can have counter-productive and unjust outcomes
• Indicates how militarisation can negatively affect rangers
• Examines the political economy of militarisation

## 1 Introduction

The rapid rise in poaching of elephants, rhinos and many other species over the last decade has prompted conservationists to think about how to respond effectively. One proposed response is a series of measures that include: more forceful or armed forms of conservation (Asiyanbi, 2016; Barbora, 2017; Massé and Lunstrum, 2016; Verweijen and Marijnen, 2018); the development and application of military style approaches (Annecke and Masubele, 2016; Büscher, 2018; Duffy et al., 2015); such as the development of informant networks, and counterinsurgency-like strategies (Adams, 2017; Büscher, 2018; Dunlap and Fairhead, 2014; Pimm et al., 2015); and the use and applications of technologies originally developed by the military (Lunstrum, 2018; Shresthra and Lapeyre, 2018). In this analysis, we term these developments the ‘militarisation of conservation’, because of the military origins and models that inform and guide these interventions.

Proponents of militarisation present the integration of military approaches with conservation practice as a necessary development (Shaw and Rademeyer, 2016; Runhovde, 2017; Henk, 2005, Henk, 2006; Hübschle and Jooste, 2017; Mogomotsi and Madigele, 2017). However, the militarisation of conservation holds potentially far-reaching consequences for practices, rhetoric, policy, and interactions between conservation actors and other stakeholders. It therefore merits attention and so here we provide an overview of an emerging body of scholarship in conservation social science that provides evidence-based critiques of the militarisation of conservation. We seek to inform and advance debates amongst academics, policy makers and practitioners. Further, while there are historical antecedents in the use of violence and militarisation to sustain conservation (Brockington, 2004; Lunstrum, 2015), we argue that in the new urgent rush to save species from extinction, many practitioners, policy makers, and proponents of current militarisation have not paid adequate attention to the potential disadvantages and long term implications of relying on such a strategy.

The body of literature we build on is firmly anchored in qualitative social sciences, and often draws on extensive fieldwork in places such as Nigeria (Asiyanbi, 2016), India (Barbora, 2017), Laos (Dwyer et al., 2015), Guatemala (Devine, 2014; Ybarra, 2016), Colombia (Bocarejo and Ojeda, 2016), Democratic Republic of the Congo (DRC) (Marijnen and Verweijen, 2016), South Africa (Büscher and Ramutsindela, 2016; Hübschle, 2017; Lunstrum, 2014), Mozambique (Massé and Lunstrum, 2016; Massé et al., 2017b; Witter and Satterfield, 2018), Tanzania (Mabele, 2016) and Central African Republic (CAR) (Lombard, 2016). Many of these studies are in-depth explorations of a single case, but there are few overviews (Duffy et al., 2015; Duffy et al., 2016; Büscher and Fletcher, 2018) or comparative studies (Lunstrum and Ybarra, 2018). Our aim in this Perspective Piece is to draw out the major themes, patterns and concerns about militarisation from the critical literature in order to inform more thorough dialogue in conservation; this synthesis of critical perspectives is intended to provide reflections in order to inform conservation policy making via the development of a clear counterpoint to the more commonly articulated position that militarisation is an appropriate, proportionate and necessary response to an urgent situation. As such, this Perspective Piece is a timely first step towards sparking a dialogue about militarisation of conservation, not least because more militarised approaches are gaining traction in other sectors such as forest protection (Asiyanbi, 2016). More thorough engagement and dialogue between different perspectives will help conservation practitioners to develop more robust and socially just policy approaches to the current poaching crisis.

First, we examine why it is important to engage critically with the militarisation of conservation. Second, we set out the main themes which characterise the emerging literature on this topic: the importance of understanding how poaching is defined, the experiences of local communities, the experiences of rangers, the challenges of conservation in areas of armed conflict, and finally the political economy of the link between conservation and militarisation. To conclude we suggest that failure to engage more critically with militarisation risks making things worse for the people involved and leads to poor conservation outcomes in the long run.

### 1.1 Why critical engagement is important

Militarised approaches to conservation appear to be expanding, becoming institutionalised and normalised in a growing number of places and amongst particular conservation non-governmental organisations (NGOs) and donors (Duffy, 2016; Marijnen, 2017; Büscher and Fletcher, 2018). Part of the reason for a shift towards militarised conservation is that some conservationists feel pressure to act urgently to prevent extinctions. This can be especially acute in conflict zones or if conservationists determine that poachers are adopting more aggressive tactics (Büscher and Ramutsindela, 2016; Marijnen and Verweijen, 2016; Kelly and Gupta, 2016; Lombard, 2016).

Of course, there are many arguments offered in favour of militarised conservation: that it is the only effective solution in an urgent situation, that conservationists need to defend themselves and the last remaining populations of endangered species against armed threats, and that use of more forceful approaches can reduce poaching leading to growing wildlife numbers, amongst others (for example see Mogomotsi and Madigele, 2017; Henk, 2005, Henk, 2006; Hübschle and Jooste, 2017; Hillborn et al., 2006; Emslie and Brooks, 1999). However, our aim is not to recount the many high profile and well-covered arguments in favour of militarised conservation. Instead our aim is to intervene in the debate by changing the focus to address important counter-arguments and concerns in more detail. While recognizing that militarisation will be varied in its details from place to place, we argue that militarised conservation as a model, even when it might result in conserving some animals and enforcing some protected areas, is fundamentally unjust. Moreover, if it is not regarded as a just and legitimate policy it will be difficult to enforce in the longer term, even with enhanced levels of resources (Wright et al., 2016).

This Perspective piece draws on research that critically engages with militarised conservation published in books, reports, and over 29 distinct journals that span a range of disciplines from geography, sociology, criminology, anthropology, political science, political ecology, conservation biology, amongst others. In addition, conservation social scientists, conservation and enforcement practitioners themselves have also pointed to the limitations of a top-down and violent approach to anti-poaching (Barichievy et al., 2017; Bennett, 2011), especially if not combined with efforts to address local socio-economic inequalities and injustices (Annecke and Masubele, 2016; Cooney et al., 2017; Haas and Ferreira, 2018). Indeed,even those who argue that militaries, such as the Botswana Defence Force (BDF), have been successful in leading anti-poaching in the country, caution against the use of military approaches in conservation (Henk, 2005, Henk, 2006).^1^

A case in point concerns the development of technological solutions, like surveillance technologies aimed at detecting poachers, as part of militarised conservation. Conservationists may find these technologies compelling, but being overly enchanted with them risks obscuring how they can waste or divert scarce resources; indeed there is a lack of transparency about the effectiveness of such technologies, the costs of which run into millions of dollars^2^ (Lunstrum, 2014; Hahn et al., 2017; Gore (2017). Furthermore, many conservationists do caution against adopting technologies that have military origins, recognizing that they can be expensive and possibly divert important investment in other aspects of conservation, and that they do not address the underlying causes of dwindling wildlife numbers (Berger-Tal and Lahoz-Monfort, 2018, 5).

Reflecting on the negative implications of militarised conservation is vitally important precisely because of its (generally) positive presentation by NGOs, international donors, and national governments. Proponents of militarised conservation often present forceful approaches as a noble or heroic quest to save species (Marijnen and Verweijen, 2016; McClanahan and Wall, 2016). From this perspective, criticism may appear as an unhelpful distraction from the urgent operational challenges faced by practitioners in the field. Critics have been portrayed as naïve, lacking in understanding, as pseudo-scientists or even as hostile towards conservation (Hübschle and Jooste, 2017; Mogomotsi and Madigele, 2017).^3^

Such portrayals obscure the nature and value of (critical) social science work on conservation (see Bennett et al., 2016; Charnley et al., 2017). As Sandbrook et al. (2013) point out, there is sometimes a false distinction drawn between those who work *on* and *in* conservation. Indeed, researchers often work closely with practitioners, and have provided an important alternative avenue for them to draw attention to and express their concerns, criticisms and frustrations (Barbora, 2017; Bennett et al., 2016; Lunstrum, 2014; Massé et al., 2017a, Massé et al., 2017b. Critical engagement can facilitate a better understanding of militarised conservation, its short and long-term implications, why alternatives are needed and what these might look like. As such, this Perspective piece aims to provide a basis for further debate and dialogue in conservation to enable conservationists to improve practices, and help develop more effective and more socially just forms of conservation. A vital first step in this is to identify and discuss the range of negative impacts of militarisation as a strategy to address wildlife poaching that are found in the literature.

## 2 Focusing on the symptoms, not the root causes of poaching

One of the criticisms that has been raised about militarised conservation is that it does not address the underlying reasons for why people engage in poaching and trafficking; instead it focuses on tackling the symptoms (poaching and trafficking) of a much deeper and complex structural contexts that is at the root of these practices (Duffy et al., 2015; Hübschle, 2017; Witter and Satterfield, 2018). Reflecting on the body of more critical scholarship on militarised conservation can help conservationists to step back and consider the wider dynamics of what produces poaching in the first place. Scholarship also shows how the portrayal and treatment of poachers as criminals could be ineffective, and even counterproductive. The history of poaching is central to this debate: there is a well-established body of literature exploring how some forms of hunting, and not others, became defined as poaching and the importance of examining how poverty, inequality, historical grievances and the continuing effects of colonial and racial discourses shape understandings of poaching (see Challender and MacMillan, 2014; Duffy et al., 2015; Hübschle, 2017; Neumann, 2004; Peluso, 1993). Similar arguments about the need to understand and address these underlying historical and structural factors to tackle environmental and wildlife crime effectively are also made by some green criminologists, such as Gore (2017) Wyatt (2013) and Cao and Wyatt (2016).

Recent scholarship on the militarisation of conservation also focuses on unpacking and analysing the portrayals of illegal wildlife trade issues by interested parties. Central to this task is the need to interrogate the discursive and visual representation of poaching. Militarised conservation is characterised by a process of ‘moral boundary drawing’ between rangers as heroes and poachers as villains (Neumann, 2004). Such boundaries are drawn through a variety of representational practices in policy literature, academic articles and fund-raising activities (Marijnen and Verweijen, 2016; Massé, 2018). These boundaries can be used to explain and justify the use of coercion and (deadly) violence against poachers (Neumann, 2004; McClanahan and Wall, 2016). There are direct parallels between the present day criminalisation of poachers and colonial era initiatives to control or outlaw hunting by African communities that produced deeply held grievances and animosity towards wildlife conservation (Duffy et al., 2015; Duffy et al., 2016; Ramutsindela, 2016). Many of these grievances still persist today and forms part of the reason for why young men might enter the poaching economy (Hübschle, 2017), as discussed in the next section.

The example of Virunga National Park in eastern DRC shows why representations *matter* for the practice of conservation. The communications team of the NGO managing the park heavily deploys hero versus villain categorisations to attract donations, for instance for the ‘Fallen Rangers Fund’. In its messaging, it consistently repeats the figure of ‘175 ranger deaths in twenty years’. Yet these numbers are presented in a decontextualised way: for example, it is rarely mentioned that people other than rangers were also killed, or that rangers were engaged in destroying homes or fields as part of their operations, (Verweijen and Marijnen, 2018; Vikanza, 2011). Instead, park communications simply portray the rangers as heroes battling against undefined armed groups, without outlining which specific group is involved and why they attack the park guards. This deliberate erasure of abuses committed by rangers gives external audiences the impression that rangers are always the heroes and contributes to a more general lack of understanding for why local communities may resist conservation initiatives (also see Massé, 2018). Identifying and tackling abuses committed by rangers (environmental and human rights abuses) is vitally important – failure to do so undermines trust between conservation authorities and people, and does a disservice to those rangers who conform to very high standards of personal conduct. In sum, moral boundary drawing between rangers as conservation heroes and evil poachers is problematic for three reasons. First, it obscures how some rangers are involved in activities that have a negative impact upon people and biodiversity (Neumann, 2004; McClanahan and Wall, 2016)–failure to recognise this, means that economic and training support for militarisation can increase the capacity to engage in such abuses (Lombard, 2016). Second, it ‘traps’ rangers in a particular role, rendering it more difficult to bring to light the complex variety of *actual* ranger stories and experiences needed to improve their working conditions and effectiveness (as discussed below). Third, by decontextualising the death of rangers, the park is able to generate more financial support for a military-style response, even though this has fostered more direct attacks by rebel groups against the park (and park rangers), ultimately leading to a cycle of violence (Verweijen and Marijnen, 2018).

### 2.1 Local communities' experiences of militarised conservation

Critical engagement with militarised conservation also means exploring and revealing the everyday challenges and problems encountered by people living in and around areas affected by militarised conservation, including the ways it can infringe upon their rights and daily lives.

Militarised conservation can mirror and recreate past injustices, which risks alienating inhabitants of conservation spaces. For example, militarised conservation tactics in specific contexts in South Africa often resemble apartheid-era counterinsurgency practices, where efforts to win the support of local people also coincide with tactics of intimidation and use of violence. These tactics also currently extend into Mozambique, and include: the development of informant networks, co-option and development of cultures of mistrust within communities (Annecke and Masubele, 2016; Lunstrum, 2015; Massé et al., 2017a, Massé et al., 2017b); raiding and invading people's homes in operations to uncover evidence of wildlife crimes (Ramutsindela, 2016; Massé et al., 2017a; Büscher, 2018); and active displacement of communities for conservation (Massé and Lunstrum, 2016; Witter and Satterfield, 2018). More forceful approaches to conservation can also be accompanied by new incentive schemes, such as the provision of game meat to schools and water reticulation programmes. However, such interventions can simply serve as stop-gap measures, or as distractions which do not address the systemic problems which produce incentives to engage in illegal hunting in the first place. There is a debate in conservation about whether it can or should address poverty and inequality; this is important because efforts to address these problems underpin ‘hearts and minds’ approaches. However, these approaches are then systematically undermined by practices of intimidation, violence and surveillance which can be part and parcel of militarisation (Massé and Lunstrum, 2016; Massé et al., 2017a; Ramutsindela, 2016). As discussed in more depth below, addressing inequalities and ensuring that conservation does not exacerbate them is necessary to tackle the underlying causes of poaching in the longer term (see for example Cooney et al., 2017; Haas and Ferreira, 2018; Hübschle, 2017; Hübschle and Shearing, 2018; Duffy et al., 2016; Duffy et al., 2015).

Local inhabitants' negative experiences of these more forceful forms of conservation are also inadequately incorporated into portrayals of rangers as heroes. When any type of conservation practice is presented as inherently good, it becomes difficult to investigate and address alleged abuses by conservation staff (Moreto et al., 2015). This can lead to a loss of accountability and legitimacy in the eyes of local people and the international community.

Despite often being crucial to the success of conservation efforts, the experiences of the people living in the areas concerned are overlooked in debates about the militarisation of conservation. Understanding what militarisation means for those people can shed light on how the drive to save species by more forceful means has counterproductive effects in the longer term. For example, one of the most significant problems with militarisation is that it has the capacity to alienate local communities who object to the use of force to protect wildlife, the development of cultures of surveillance and their continued (often violent) exclusion from protected areas; such approaches will lose the support of the very people who are central to conservation efforts in the longer term (Duffy et al., 2015; Cooney et al., 2017; Hübschle, 2017; Massé et al., 2017a, Massé et al., 2017b). Further, a recent study by Holden et al. (2018) details emergent findings that demand reduction campaigns coupled with sustainable livelihood approaches are more effective at tackling poaching for ivory than enhanced policing and enforcement alone. This further calls into question the long-term sustainability of militarised approaches.

### 2.2 The experiences of rangers

There is also a need to gain a better understanding of the *lived experiences* of those involved in implementing militarised conservation, notably rangers. A growing number of researchers are engaged in this task (Massé et al., 2017a, Massé et al., 2017b; Moreto, 2015a, Moreto, 2015b; Moreto et al., 2015; Moreto et al., 2017; Gore, 2017). Not claiming to speak for rangers, these researchers instead offer avenues to draw attention to the problems and challenges that rangers face, and *their* own concerns and criticisms of militarised conservation. In so doing, they assist with and support documenting ranger concerns, and act to amplify them. This is important work because rangers operate in complicated power hierarchies, and often fear that they will lose their jobs if they criticise conservation authorities, so they cannot necessarily speak out publicly (Annecke and Masubele, 2016; Massé et al., 2017a, Massé et al., 2017b; Moreto, 2015a, Moreto, 2015b). Moreover, understanding rangers' experiences relates directly to an understanding of their practices in the field and their motivations, as the recent *WWF Ranger Perception* surveys show.^4^

The idea that rangers do what they do simply because they love nature, and that they willingly engage in hard-line approaches to anti-poaching does not map well on to their actual experiences of carrying out the practices of militarised conservation (Moreto, 2015a, Moreto, 2015b).^5^ For example, there are rising rates of Post-Traumatic Stress Disorder (PTSD) amongst rangers in Kruger National Park, and there are reports of many more staff diagnosed with other stress-related conditions.^6^ We also see a re-direction of ranger duties away from ‘their typical role as conservationists to become active players in guerrilla warfare, putting their lives in constant jeopardy’.^7^ Indeed, rangers are increasingly targets of violence themselves (Lunstrum, 2014; Massé et al., 2017a, Massé et al., 2017b), which is especially harsh given that militarised anti-poaching may not be what they signed up for when they entered the profession (Annecke and Masubele, 2016).

The entire spectrum of ranger experiences is important. Rank-and-file staff involved in militarised conservation often express concern about what they are expected to do, and there can be a (perceived or genuine) lack of transparency of senior staff and the wider institutions engaged in designing and implementing militarised conservation (Moreto et al., 2015). One reason for such opacity is that conservationists face immense pressures to demonstrate that they are making in-roads against poaching. In the Kruger National Park, key performance assessments of management staff are narrowed to reducing the rate of rhino killed per day, irrespective of the pressures faced by conservation managers. Such pressures have snowballing effects that can lead to the use of excessive force, torture, and even extra judicial killings of suspects, as is well documented in Tanzania (Carlson et al., 2015; Mabele, 2016). The negative effects of this pressure also extend to conservation staff themselves, as a culture of suspicion and mistrust can lead to toxic work environments, increasing workplace stress. There is often an unacknowledged racial politics running through conservation circles as well. Conscious and unconscious bias amongst white staff, which leads to unfair and incorrect assumptions about fellow black staff prevents the development of effective working relationships. This undermines and unravels any gains made in terms of increasing diversity and equality in the workplace in the longer term (Moreto, 2015a, Moreto, 2015b; Mbaria and Ogada, 2016).

Rather than reducing rangers to a singular category of ‘conservation heroes’ it is important to highlight and understand the complex realities faced by rangers in order to improve their well-being and working conditions (Massé et al., 2017a, Massé et al., 2017b; Moreto, 2015a, Moreto, 2015b).^8^ Yet, many aspects of ranger experiences remain understudied, including: How do rangers regard the use of tracking technology, which monitors their movements during the working day? What are the implications of such work place surveillance for labour relations? Are rangers paid adequately and on time? Do rangers feel they have the right equipment, and are there sufficient and appropriate pathways through the profession? What are their other options for employment? What kinds of pressures do their families face? Addressing these questions requires thorough and sustained research from the social sciences, and could benefit from developing an analysis which is more firmly anchored in debates about labour relations rather than conservation per se.

### 2.3 Conservation and armed conflict

One of the strongest arguments in favour of militarised conservation is that it is the best (or only) workable option in areas of intense armed conflict. However, the intersections between conservation and wider dynamics of armed conflict remain ill understood. Yet there is an emerging body of work, often drawing on insights from a range of academic disciplines such as development, and peace and conflict studies, that specifically examines how militarised conservation intersects with conflicts and violence. (Lombard, 2016; Verweijen and Marijnen, 2018).

In this Perspectives piece we argue that militarisation of conservation is in itself fundamentally problematic precisely because it can serve to embed conflict dynamics further, rather than resolve them (Marijnen, 2018). A pertinent question which requires further debate is whether militarised conservation ultimately contributes to rising levels of violence in contexts of armed conflict. When conservationists operate in conflict zones, they often face intense pressures and can feel directly threatened by armed groups and by heavily armed poachers. When faced with such threats it can seem a ‘common sense’ response for rangers to resort to the use of force to protect wildlife and themselves. However, when readily using force, it may occur that groups (including rangers), which are armed for conservation purposes, are simply regarded as another armed group engaged in a conflict. This can lead to an escalation in arms and in levels of violence, and once such a dynamic is generated it is difficult to de-escalate (Duffy, 2016; Humphreys and Smith, 2014).

Another key issue is the well publicised and high profile claims that poaching is a crucial funding strategy for militias, rebel groups and even terrorist networks. While conservation social scientists do not deny that poachers may be heavily armed in certain places, especially in conflict zones, they do examine and question the claims that there is a clear link between illegal wildlife trade and funding for armed conflict. For example, the Elephant Action League controversially claimed Al Shabaab use ivory to fund terrorist activity. This narrative was taken up by a range of NGOs, philanthropic foundations, governments and media outlets (Duffy, 2016). However, several studies show that these claims are poorly evidenced and are based on false assumptions (Duffy, 2016; Smith & Haenlein, 2016; Sommerville, 2016; White, 2014; Kelly et al., 2018). While there has been a recent move away from making such explicit claims about Al Shabaab because of the lack of evidence, it has not stopped the circulation of the problematic narrative that ivory (and illegal wildlife trade more generally) is used to fund conflict and specifically militant groups such as Janjaweed, the Lord's Resistance Army and Boko Haram (Kelly et al., 2018). For example, Achim Steiner when he was head of UNEP, Prince William and the #Whosesideareyouon campaign of United for Wildlife, Wildlife Conservation Society's 96 Elephants Campaign and Conservation International's Direct Connection campaign all drew links between (broadly defined) international terrorism and the illegal wildlife trade (Duffy, 2016; Sommerville, 2016). Even while lacking evidence, such claims increase the sense of urgency to save species in the face of intense or growing pressures of armed conflict, and are thus used to justify a shift towards more militarised forms of conservation.

Increasing collaborations between conservationists, national armies and UN Peace Keeping Operations (UNPKO) could also benefit from greater critical reflection. This could draw on long standing debates in politics and international relations about the risks and challenges of working with these kinds of military institutions. For instance, much of the literature on UNPKOs highlights that peacekeeping and peace enforcement often means taking sides, and that it is not possible (or even necessarily desirable) to be ‘neutral partners’ in a conflict zone (Verweijen, 2017; Fassin, 2012). This is an important point for conservationists seeking to partner with UNPKOs: working with military actors means becoming more deeply embedded in the very conflict dynamics that undermine conservation efforts.

Collaborations between military actors and conservationists might also spark tensions: military actors are trained in a particular type of approach and rules of engagement that are geared towards overlapping areas of defence, counter-insurgency and pursuing warfare. This approach differs from the role and purpose of conservationists. It cannot be assumed that government forces have a clean record on conservation issues either. In specific places, there have been accusations of direct involvement of military personnel in poaching or other forms of illegal and damaging natural resource extraction, even using poaching to fund their operations (Carlson et al., 2015; Ellis, 1994; Titeca, 2013). There are also risks in providing military training and equipment to rangers because there are instances where the new skills and weaponry are turned back on wildlife and local communities. This occurred for example in CAR where a park guard who received paramilitary training funded by the European Commission became a rebel leader with the Seleka movement, and many of the other externally trained park guards joined as well (Lombard, 2016).

Conservationists also need to be aware of the human rights record of their collaborators. In some instances, soldiers from national armies engage in human rights abuses and are regarded as a repressive and hostile force. Working with military partners can therefore contribute to a more negative view of the work of park rangers amongst those who bear the brunt of these abuses (Verweijen and Marijnen, 2018). Collaborating with military partners with records of social and ecological abuses is potentially problematic to conservationists because it can inflict significant reputational damage on the international stage, thereby undermining international support for conservation. These considerations highlight the importance of studying military collaborations and the effects on conflict dynamics in zones of armed conflict, in particular when conflict dynamics are central to justifications for the need to militarise conservation in the first place.

### 2.4 The political economy of the militarisation of conservation

One central, but often overlooked, question in arguments about militarisation of conservation is: who wins and who loses from increasing militarisation? Who profits from the development of more militarised approaches, and how does that shape or produce specific conservation strategies? Answering these questions requires examining the political economy of militarisation of conservation.

As a growing number of studies show, the militarisation of conservation can be driven by the demand for profits from private sector actors seeking to expand into new markets. For example, Devine (2014) examines the intersections of ecotourism, conservation and militarisation in Guatemala, drawing out the ways in which ecotourism development has become a means by which the Guatemalan state has revived and repurposed tactics of counterinsurgency warfare derived from the country's civil war. Massé and Lunstrum (2016) argue that anti-poaching strategies in and around Kruger National Park constitute ‘accumulation by securitisation’ whereby anti-poaching security and training offers lucrative avenues for the private sector (Lunstrum, 2018). Such accumulation is evident in the number of Private Security Companies (PSCs) offering their services for training or for direct engagement in anti-poaching operations. Critics of using PSCs in conservation point out that it is important to examine past examples of poor practice. For example, Neumann (2004) discusses the case of Liwonde National Park in Malawi, where rangers trained by a PSC were implicated in human rights abuses over a two-year period. In the past, a number of conservation NGOs have been engulfed in scandals involving intelligence gathering for anti-poaching via contracting private companies staffed by ex-special forces. One of the best known examples is Operation Lock during the mid 1980s, which was carried out by KAS Enterprises (a private military company). While intended to conduct wildlife crime sting operations, it was later revealed that they also gathered intelligence on anti-apartheid activists for the South African state. As WWF-International had provided funds to this operation, the NGO's reputation was damaged (Rademeyer, 2012). Despite these negative experiences, several conservation NGOs today continue to hire private intelligence companies staffed by former operatives from intelligence services, including the former Bureau of State Security (BOSS) and Mossad (Massé et al., 2017a).

The concerns raised about the role of PSCs and partnering with intelligence specialists, especially external or ‘foreign’ operatives, are shared by professional bodies. In 2017, the Game Rangers Association of Africa issued a statement expressing concerns about the activities of security agencies from outside Africa. They noted these agencies' lack of coordination, lack of understanding of the operating environment, lack of ecological sensitivity, lack of knowledge of the legal frameworks that rangers operate in as well as the lack of proper vetting of foreign security agents and profiteering by military equipment manufacturers.^9^

Conservation NGOs themselves are also becoming more involved in surveillance, intelligence gathering and developing informant networks (Massé et al., 2017a, Massé et al., 2017b; Sandbrook, 2015). The risks related to these practices are not sufficiently acknowledged by their proponents. For instance, NGO staff may not have the training to collect and store sensitive data securely in order to ensure that participants or informants are not put at increased risk (although there is now more attention on this theme).^10^ Additionally, the process of gathering information may itself be problematic if informants feel physically threatened or fear losing their jobs. Furthermore, in certain areas where it is practiced, intelligence-led approaches to conservation have already fomented intra-community tensions resulting in violent attacks (Biggs et al., 2016). Practices of intelligence gathering by NGOs and private security sector partners are embedded in and shaped by the attitudes and approaches of the individuals and organisations involved in it, which may not align with local attitudes (Massé et al., 2017a, Massé et al., 2017b; Roe et al., 2015).

Another dimension of the political economy of militarised conservation is that this practice can become commodified in itself. For instance, it may become a form of ‘spectacle’ that is central to fundraising efforts by enticing ‘consumers’ to directly fund armed conservation efforts. An example is the multi-media campaign launched by the Virunga Foundation after the release of ‘Virunga, the movie’ whereby the viewer is asked to become part of ‘Virunga's epic fight’ by donating money, for instance to fund patrols or dog-tracking teams, without understanding the on the ground effects of these strategies in this conflict zone (Marijnen and Verweijen, 2016).

The redistribution of resources, attention, and focus resulting from green militarisation and the political economy of fundraising that has emerged around it also has broader ecological impacts. This is especially the case in areas where (para)military actors are at the helm. Massé (2018), for example, demonstrates how many conservationists in areas where former and current military personnel have increasing decision-making power are concerned about the impacts this has on broader conservation activities. Similar concerns are voiced with regards to shifts in fundraising practices that focus on simplistic understandings of poaching and the responses needed. The result is an increasing allocation of scarce resources to (para)militarised enforcement approaches, and away from other conservation priorities that may be less spectacular, but no less important. Indeed, officials from South African National Parks draw attention to how rangers in Kruger spend 90% of their time hunting potential rhino poachers at the expense of their more traditional conservation management and monitoring roles like basic ecological and landscape monitoring assessments (Annecke and Masubele, 2016; Hübschle and Jooste, 2017). This is coupled with a shift in ranger training away from holistic conservation and ecological management towards more narrow paramilitary and counter-insurgency tactics (Ibid.; Lunstrum, 2014). Our research indicates similar trends in Mozambique and elsewhere in Sub-Saharan Africa. This is a worrying development for the broader state of biodiversity conservation now and in the long term, as resources are diverted away from activities that are essential to the ecological integrity of protected areas. Therefore, more research into how the trend of conservation's militarisation risks jeopardizing the broader ecological health of protected areas is needed.

## 3 Conclusion

It is vitally important to reflect on militarised actions and interventions in conservation. Failure to do so, especially in urgent situations, can lead to a greatly enhanced willingness to use violence, with counterproductive and unjust outcomes for people and for wildlife. The sense of urgency produced by concerns that wildlife poaching and trafficking will lead to extinctions has led to the argument that there is a need to act before it is too late. Moreover, this urgency feeds into appeals that the *ends* (saving species) justify the *means* (use of force, including deadly force). However, conservationists must not simply accept this as a stark ‘no choice’ pathway for tackling trafficking and poaching. Amidst the sense of urgency to save wildlife and prevent extinctions via any means necessary, those who favour and support militarisation have not paid enough attention to how it will fundamentally reshape conservation in the longer term; in this Perspective piece we have cast light on the wider dimensions of, and potential problems with, relying on a militarised approach.

In order to conserve species and develop socially just and sustainable strategies we need critical, imaginative, and often uncomfortable thinking to get us out of the ‘urgency’ of the moment and put things, literally and figuratively, in perspective. This Perspectives piece is intended as a first step towards that: the negative effects of militarisation and the criticisms of it need to be made clear in order to build effective alternative approaches. Developing such alternatives also means that it is essential that we develop an understanding of all the steps that lead to the specific moment when rangers encounter poachers, think about how and why people engage in poaching and what the effects of using forceful strategies are on rangers, their families and wider social networks. In addition, we should further analyse the political economy of militarised conservation, learn lessons from the past (including the colonial legacies that produced poaching), think through the challenges of conservation in contexts of armed conflict and consider how rangers and communities in and around protected areas experience militarisation. Further research inspired by this kind of thinking can facilitate the design of conservation policies that aim to conserve species in ways that are sustainable, effective and locally acceptable.

Failure to engage more critically with militarisation will make things worse for the people involved, and could lead to poor conservation outcomes as well. The use of forceful and violent strategies in conservation can be counterproductive and can lock conservationists into an escalation of violence, a dynamic that also risks undermining other conservation priorities. Yet, we only have a partial grasp on the full range of implications of the militarisation of conservation, and its intersections with broader social, political and economic contexts. Social sciences (as well as Arts and Humanities) researchers therefore have much to offer in developing future research on militarised conservation. Such research provides important opportunities for dialogue to develop better conservation practice, with more positive outcomes for wildlife and for people.

## Footnotes

1 It should be noted that despite these claims of success, by 1995, 8 years after the BDF took over anti-poaching, the black rhino was declared locally extinct and there were only 20 white rhinos left (Emslie and Brooks, 1999).

2 http://www.defenceweb.co.za/index.php?option=com_content&view=article&id=47259:no-more-drones-for-kruger&catid=35:Aerospace&Itemid=107

3 https://news.mongabay.com/2017/10/attacks-on-militarized-conservation-are-naive-commentary/, accessed 07.10.19.

4 https://c402277.ssl.cf1.rackcdn.com/publications/880/files/original/Ranger_Perception_Africa_%28FINAL%29.pdf?1464013250

5 Ibid.

6 http://www.gameranger.org/what-we-do/projects/140-ranger-wellbeing-project.html; https://www.scientificamerican.com/article/the-trauma-of-saving-animals/; https://news.nationalgeographic.com/news/2014/06/140627-congo-virunga-wildlife-rangers-elephants-rhinos-poaching/

7 http://www.gameranger.org/what-we-do/projects/140-ranger-wellbeing-project.html

8 https://c402277.ssl.cf1.rackcdn.com/publications/880/files/original/Ranger_Perception_Africa_%28FINAL%29.pdf?1464013250

9 Full text of the statement can be found at http://www.gameranger.org/news-views/media-releases/170-media-statement-the-use-of-military-and-security-personnel-and-tactics-in-the-training-of-africa-s-rangers.html (accessed 08.01.18).

10 http://pdf.usaid.gov/pdf_docs/pa00m3r4.pdf
